# Medicare-Medicaid Dual Eligible Special Needs Plan Enrollment in Maryland

**DOI:** 10.1093/geroni/igaf122.3503

**Published:** 2025-12-31

**Authors:** Christine Gill, Roberto Millar, Christin Diehl

**Affiliations:** University of Maryland, Baltimore County, Baltimore, Maryland, United States; University of Maryland, Baltimore County, Baltimore, Maryland, United States; University of Maryland, Baltimore County, Baltimore, Maryland, United States

## Abstract

Nationally, health care spending for Medicare-Medicaid duals is higher than for non-duals in either program. Programs integrating care across these separate programs—one type being dual eligible special needs plans (D-SNPs), a Medicare Advantage (MA) plan—produce mixed results on improving care coordination and reducing costs. This retrospective cohort study examined sociodemographic and geographic factors associated with 2021 D-SNP enrollment among duals in Maryland—a state with low MA enrollment. We report descriptive statistics by Medicare program type (D-SNP or Traditional Medicare [TM]) and logistic regression analyses. Among 109,646 duals, 9% were enrolled in D-SNPs, and 60% were 65 years or older. We found regional differences: Baltimore City enrollees were 1.6 (95% confidence interval (CI) 1.5-1.7) times more likely than those in Central Maryland to select D-SNPs compared with TM, and neighborhoods with the highest quintile of disadvantage had 1.4 (95% CI: 1.3-1.5) times the likelihood of selecting D-SNPs compared with TM. Full duals were less likely to select D-SNPs over TM compared with partial duals (0.8 [95% CI: 0.7-0.8]); those 65 years and older were more likely compared with younger (1.6 [95% CI: 1.5-1.7]); and Black (2.2 [95% CI: 2.1-2.3]) and Latino (1.6 [95% CI: 1.4-1.8]) were more likely compared with White duals. Given that racial and ethnic minorities and those from more disadvantaged neighborhoods are enrolled in D-SNPs disproportionately in Maryland, additional research should be conducted to examine whether health outcomes and care quality are equitable across programs to inform policy integrating care for this high-needs population.

